# Targeting Acetylcholinesterase: Identification of Chemical Leads by High Throughput Screening, Structure Determination and Molecular Modeling

**DOI:** 10.1371/journal.pone.0026039

**Published:** 2011-11-30

**Authors:** Lotta Berg, C. David Andersson, Elisabet Artursson, Andreas Hörnberg, Anna-Karin Tunemalm, Anna Linusson, Fredrik Ekström

**Affiliations:** 1 Department of Chemistry, Umeå University, Umeå, Sweden; 2 Swedish Defence Research Agency, CBRN Defence and Security, Umeå, Sweden; University of Queensland, Australia

## Abstract

Acetylcholinesterase (AChE) is an essential enzyme that terminates cholinergic transmission by rapid hydrolysis of the neurotransmitter acetylcholine. Compounds inhibiting this enzyme can be used (*inter alia*) to treat cholinergic deficiencies (e.g. in Alzheimer's disease), but may also act as dangerous toxins (e.g. nerve agents such as sarin). Treatment of nerve agent poisoning involves use of antidotes, small molecules capable of reactivating AChE. We have screened a collection of organic molecules to assess their ability to inhibit the enzymatic activity of AChE, aiming to find lead compounds for further optimization leading to drugs with increased efficacy and/or decreased side effects. 124 inhibitors were discovered, with considerable chemical diversity regarding size, polarity, flexibility and charge distribution. An extensive structure determination campaign resulted in a set of crystal structures of protein-ligand complexes. Overall, the ligands have substantial interactions with the peripheral anionic site of AChE, and the majority form additional interactions with the catalytic site (CAS). Reproduction of the bioactive conformation of six of the ligands using molecular docking simulations required modification of the default parameter settings of the docking software. The results show that docking-assisted structure-based design of AChE inhibitors is challenging and requires crystallographic support to obtain reliable results, at least with currently available software. The complex formed between C5685 and *Mus musculus* AChE (C5685•*m*AChE) is a representative structure for the general binding mode of the determined structures. The CAS binding part of C5685 could not be structurally determined due to a disordered electron density map and the developed docking protocol was used to predict the binding modes of this part of the molecule. We believe that chemical modifications of our discovered inhibitors, biochemical and biophysical characterization, crystallography and computational chemistry provide a route to novel AChE inhibitors and reactivators.

## Introduction

**Figure 1:**
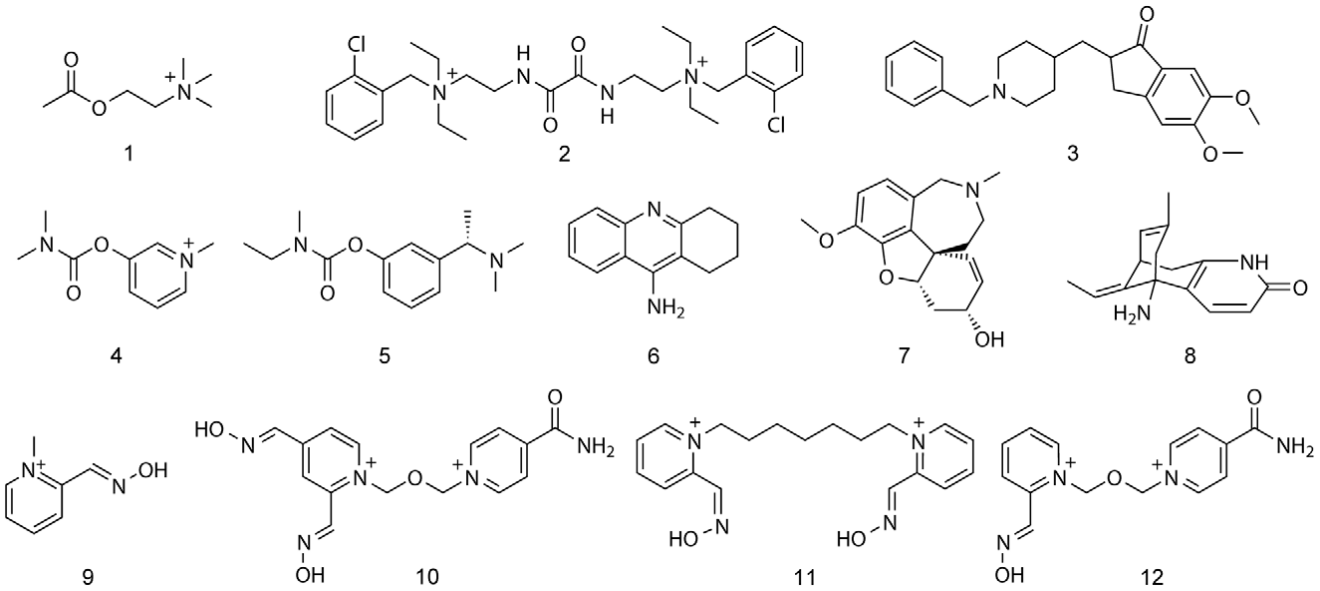
Chemical structures of ligands that bind to AChE. The native substrate acetylcholine **1**; inhibitors, ambenonium **2**, donepezil **3**, pyridostigmine **4**, rivastigmine **5**, tacrine **6**, galanthamine **7** and huperzine A **8**; nerve agent antidotes, 2-PAM **9**, HLö-7 **10**, ortho-7 **11** and HI-6 **12**.

The cholinergic system controls signals from nerve cells to muscle cells or other nerve cells and is essential in all higher organisms. The neurotransmitter acetylcholine **1** (ACh, Figure 1) modulates the signaling in pre- and post-synaptic cells in the synaptic cleft through its release and subsequent binding to the cholinergic receptors. Acetylcholinesterase (AChE) is an essential enzyme anchored to the cell membrane close to the cholinergic receptors, which effectively terminates cholinergic transmission by rapid hydrolysis of ACh [1]. AChE is found both in the peripheral nervous system (PNS) and the central nervous system (CNS).

The crystal structure of AChE shows that the catalytic triad, which is formed by serine, histidine and glutamate, is located at the bottom of a narrow 20-Å-deep gorge that penetrates halfway into the enzyme and widens close to its base [2], [3]. The ligand-binding cavity is lined with aromatic residues that account for approximately 40 percent of the cavity surface. The entrance of the gorge is termed the peripheral anionic site (PAS) as it was initially believed to contain several negatively charged amino acids due to its preference of binding cationic ligands [4]. However, the crystal structure indicates that insufficient acidic amino acids are located close to the ligand-binding cavity to support this hypothesis. Instead it has been shown that aromatic residues interact with cationic ligands [2], [5]. A similar interaction pattern can be seen at the catalytic site (CAS), and it is believed that ACh initially binds to the PAS and then rapidly diffuses down to the catalytic site [1], [2].

Compounds that inhibit AChE are very powerful drugs and toxins due to the essential function of the enzyme. In drug discovery programs, AChE inhibitors are of great interest for treatment of cholinergic deficiencies in the PNS (e.g. myasthenia gravis) and CNS (e.g. Alzheimer's disease). In contrast, some of the most dangerous toxins currently known are AChE inhibitors, for example the green mamba (*Dendroaspis angusticeps*) toxin fasciculin, and the nerve agent sarin. In general, most AChE inhibitors mimic ACh by possessing a quaternary amine or a basic nitrogen, hence they are positively charged species at physiological pH and can form cation - aromatic interactions with AChE. Examples of such inhibitors are the drugs ambenonium **2** used to treat myasthenia gravis, and donepezil **3** used to treat Alzheimer's disease. In addition, some inhibitors mimic the hydrolytic center (i.e., the ester bond of ACh) by other functional groups (e.g., carbamates and organophosphorus compounds; OPs). These compounds inhibit AChE by forming a covalent bond with the catalytic serine residue that is either reversible, as for carbamate containing drugs, or irreversible, as for most OPs. The carbamate moiety has been, and still is, a key functional group in medicinal chemistry programs to develop drugs targeting AChE, for example the drugs pyridostigmine **4** (myasthenia gravis) and rivastigmine **5** (Alzheimer's disease).

Many of the AChE inhibitors and lead structures known today are derived from natural products, for example tacrine **6**, galanthamine **7** and hyperzine A **8** [6]–[8]. However, the molecule that was further developed into donepezil **3** was discovered through random screening of a compound collection [9], [10]. Another important group of small organic compounds that interact with AChE are oxime-based reactivators, such as pralidoxime **9** (2-PAM), HLö-7 **10** and Ortho-7 **11** [11]–[13]. These compounds can reactivate otherwise irreversibly inhibited AChE (e.g., by OPs) via a nucleophilic attack that cleaves the covalent bond between the enzyme and the OP adduct. The chemical structures of the available antidotes are generally similar, differing only in the number of pyridinium rings (mono or bis), the length of the central linker, and the position of the nucleophilic oxime on the pyridinium ring.

The urgent need for new drugs to treat cholinergic disorders like Alzheimer's disease [14], and the limited applicability of the currently available antidotes to nerve agent intoxications (in terms of blood-brain barrier permeability [15] and spectrum [16]) make the research for new AChE inhibitors highly relevant. In the design of novel compounds, structure-based design, such as molecular docking, could be applied based on available crystal structures of AChE in complex with inhibitors or reactivators.

Most crystal structures of AChE-ligand complexes that have been obtained to date are for complexes of *Torpedo californica* AChE (*Tc*AChE) with natural products (e.g., galanthamine **7** [17] and hyperzine A **8** [18]) or oximes with *Mus musculus* AChE (*m*AChE) (e.g. Ortho-7 **11** [19] and HI-6 **12** [19], [20]). In addition, donepezil **3** has been determined in complex with *Tc*AChE [21]. On the sequence level, the main difference between the catalytic sites of the *Torpedo* and *Mus musculus* enzymes is the substitution of Phe330 in *Tc*AChE by Tyr337 in *m*AChE, the latter being similar in this respect to the *Homo sapiens* AChE (*h*AChE) [22].

Molecular docking simulations have been successfully used in many medicinal chemistry projects making them valuable tools in drug discovery [23]. However, reproducing the bioactive conformation of AChE ligands, as determined by X-ray crystallography, using molecular docking programs has proved to be problematic [24]–[26]. The results of previously published studies were heavily dependent on the type of ligand, the protein conformation, and the presence of water. Despite these difficulties, molecular docking of AChE ligands has been applied in numerous cases to explore the AChE activity of synthesized compounds in terms of protein-ligand interactions and structure-activity relationships (SARs) based on pose predictions [27]–[30].

Most of the published structure-based virtual screens to identify new AChE inhibitors have been based on simulations in which enrichments of known inhibitors have been monitored [31]–[37]. In general, these studies have found poor, or no enrichments of compounds binding to AChE [32]–[36], illustrating the challenges associated with docking of AChE inhibitors. Further, among the cases where some enrichments have been detected [32], [33], [35] there is no agreement regarding the optimal docking program. However, a successful structure-based virtual screen of commercially available compounds using flexible docking that resulted in the identification of novel AChE inhibitors has been reported [31].

In this paper, we present an *in vitro* high throughput screen (HTS) of a large collection of molecules intended to identify novel AChE inhibitors. The binding modes of a selection of the discovered hits have been determined by X-ray crystallography and reproduced by molecular docking simulations. The potential of using the hits as lead structures for AChE inhibitors and reactivators in drug discovery programs is also discussed.

## Results

### High throughput screening and determination of *IC_50_* values

**Figure 2:**
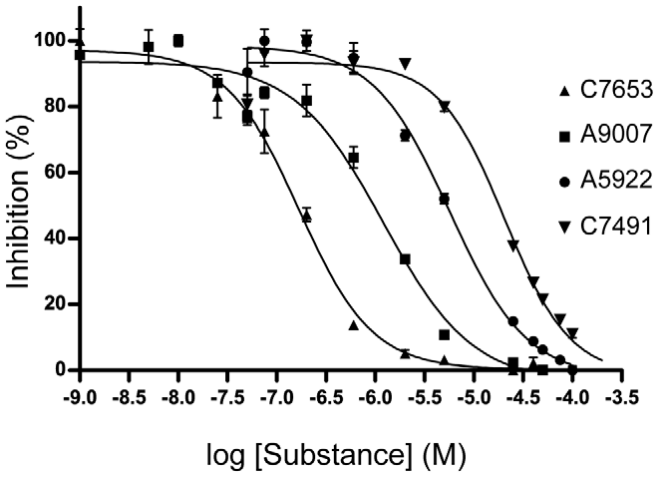
Dose-response curves for a subset of hits. In the figure, the mean ± standard deviation of 2–4 determinations is plotted.

A chemical library consisting of 17 500 substances was screened using the colorimetric Ellman assay and recombinant *h*AChE. The hydrolysis of acetylthiocholine iodide was monitored and the average slope of the positive controls was set to 100% activity. At an assay concentration of 50 µM, 124 compounds reduced the enzymatic activity of *h*AChE by at least 70% in the single replicate assays. To confirm the activity of the hits, the half-maximal inhibitory concentration (*IC_50_*) was determined for 30 compounds (Set 1, Table 1). Molecules in Set 1 were selected to represent the chemical space according to a Principal Component Analysis (PCA) of the hits' structural and physicochemical features (see section *Chemical space of AChE inhibitors*, below). The *IC_50_* determinations confirm that the 30 compounds in Set 1 are indeed AChE inhibitors, with *IC_50_* values ranging from 0.29–82 µM (Table 1 and Figure 2). Furthermore, a second data set (Set 2) of 30 substances showing activities of 0±5% in the single replicate assays was selected from neighboring positions in the established chemical space. Quadruple sampling at a ligand concentration of 50 µM confirmed that the substances in Set 2 were non-binders, but one of the substances inhibited AChE by 71%, close to the original cut-off value, and had an *IC_50_* value of 48 µM (see File S1).

**Table 1:** *IC_50_* values of the representative set of active compounds identified in the HTS.

	Set 1	
No.	Compound	*IC_50_* 1 (µM)
1	A6035	0.29±0.02
2	A7966	0.38±0.02
3	A9007	1.2±0.1
4	A7686	1.4±0.1
5	A5388	1.5±0.3
6	A7528	1.8±0.1
7	A5315	1.9±0.4
8	A7372	1.9±0.7
9	A7998	2.9±0.5
10	A5846	3.1±0.9
11	A6623	3.3±1.8
12	A6164	3.5±0.9
13	A7175	3.5±1.2
14	A5918	3.6±0.8
15	A9005	3.9±0.3
16	A5130	5.3±0.3
17	A5744	6.0±4.3
18	A5922	6.2±0.8
19	A5332	6.8±3.0
20	A7988	7.1±0.6
21	A6144	11.6±0.9
22	A6386	13.3±2.2
23	A6676	14.1±1.4
24	A6659	25.7±5.0
25	A5319	30.2±1.3
26	A6435	40.8±16.5
27	A5320	43.4±12.0
28	A7124	47.6±27.6
29	A6642	50.9±3.4
30	A7695	81.8±23.9

1 Mean ± standard deviations of 2–4 determinations.

### Crystallographic studies of selected hits

The number of hits prompted us to investigate the success rate of complex formation using a standardized protocol with no compound-specific optimization of soaking conditions. In total, 36 hits were subjected to complex-formation trials where the standardized protocol (see *Materials and Methods*) was reproduced to the best of our ability. To facilitate this approach, the *Mus musculus* AChE (*m*AChE; 88% sequence identity and all residues in the active site identical with the human enzyme) was used. The low salt and neutral pH conditions used for the crystallization of *m*AChE (see *Materials and Methods*) and the corresponding crystal packing has proven robust and suitable for studies of PAS binding ligands [20], [38].

Typically, several soaking attempts were required before a crystal displaying satisfactory diffraction was identified, hence there were more than 200 individual soaking trials in total. Interestingly, one compound resisted our efforts and repeatedly cracked the crystals during the soaking procedure, possibly indicating that the ligand induces a structural change that is incompatible with the crystal packing. In total, 35 data sets were collected and after initial refinements, electron density features that could be attributed to a small molecule were found in nine datasets. These datasets were subjected to complete structural refinement, during which two were discarded due to difficulties to accurately and conclusively build the ligand in the electron density maps. Out of the original 36 compounds, structures for seven were finalized. One of the determined structures revealed that a complex between *m*AChE and a degradation product of the compound present in the chemical library had been formed. This complex is not further discussed herein.

**Figure 3:**
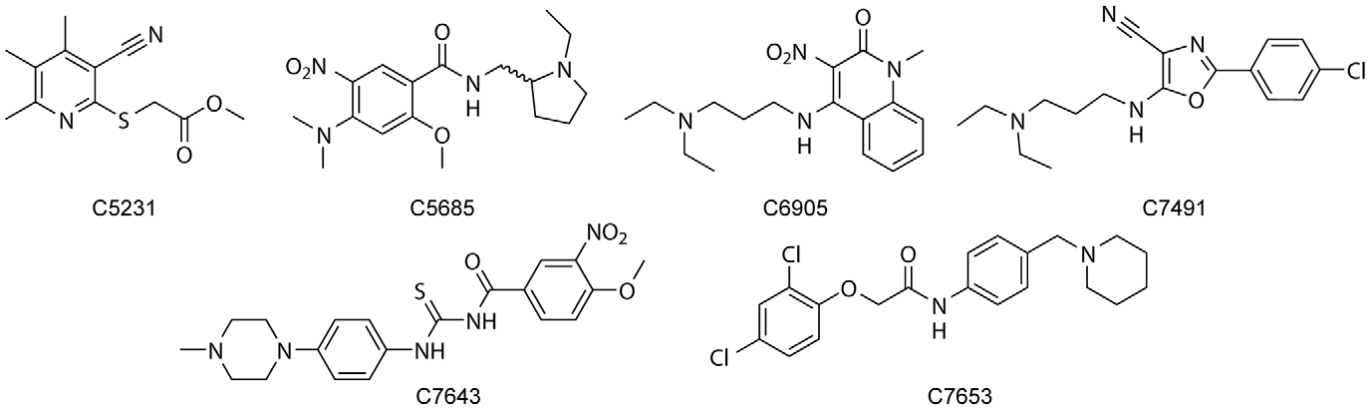
The chemical structures of the AChE inhibitors. The compounds C5231–C7653 were identified as hits in the HTS and their bioactive conformations were determined in complex with *m*AChE by X-ray crystallography.

**Figure 4:**
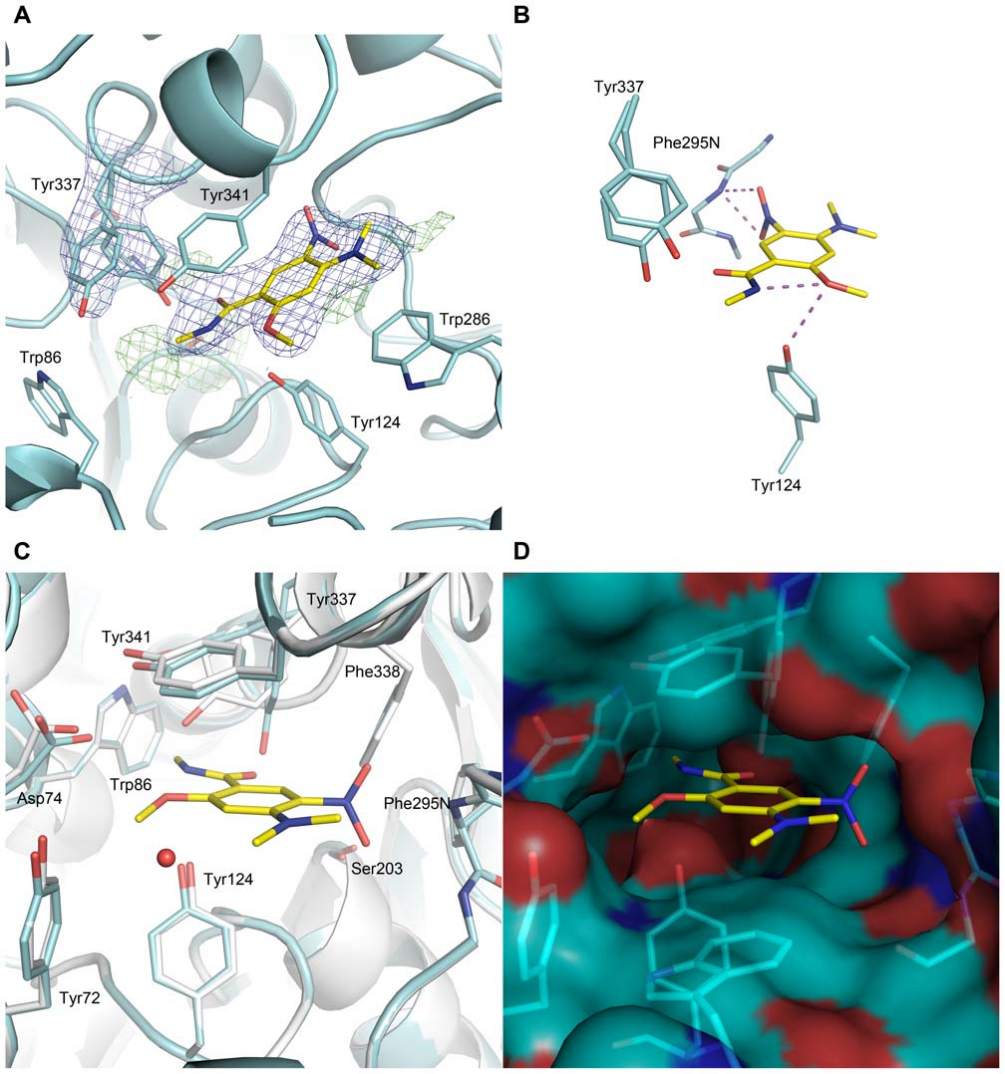
The *m*AChE binding site of C5685 determined by X-ray crystallography. The final 2*|Fo|*−|*Fc*| (contoured at 1σ) electron density maps of C5685 (yellow) and the two conformers of Tyr337 in *m*AChE (cyan) (A). Close-ups showing putative hydrogen bonding interactions (B). The binding site of C5685 at the PAS of *m*AChE; in (C), the *apo m*AChE structure (grey) has been superimposed on C5685•*m*AChE, and in (D) the Connolly surface is visualized (cyan carbon; dark blue nitrogen; red oxygen).

The crystal structures of the complexes formed by *m*AChE and the six compounds (Figure 3) were refined to a resolution ranging from 2.3 to 2.8 Å. The *IC_50_* of the successfully determined compounds ranged between 0.2 and 36 µM (Table 2). Overall, the structures are similar regarding protein backbone, side chains (the amino acids with the largest variations in the active site gorge were Tyr337 and Tyr72 with maximum atom deviations of 2.15 Å and 1.82 Å, respectively), and binding site occupancy of the ligands. One of the complexes (C5685•*m*AChE) differed slightly from the others as Tyr337 and the CAS binding portion of the ligand were disordered. The structures show extensive interactions between the ligands and amino acids in the PAS (Table 3). The ligands stack with Tyr341 and/or Trp 286 and a direct hydrogen bond between the ligand and main-chain nitrogen of Phe295 is present in five cases and in one structure the hydrogen bond is bridged by a water molecule. One of the compounds is PAS specific (C5231), while the remaining ligands have additional interactions with Tyr124, Tyr337 and Trp86 in the active site gorge and the catalytic site (Table 3). A SAR analysis based on the structures and their corresponding *IC_50_* values showed, as expected, that larger molecules with favorable interactions in both PAS and CAS (i.e., C7653 and C7645) give a stronger inhibition than ligands of moderate size and fewer interaction points (i.e., C7491 and C5231). The complex between *m*AChE and the racemate C5685 (C5685•*m*AChE Figure 4) is a representative structure for the general binding mode described above while also containing unique features, and will therefore be described in detail. The crystal structure of C5685•*m*AChE was refined to a resolution of 2.4 Å (Table 4), showing an overall structure that is very similar to the *apo* structure of *m*AChE (pdb entry code: 1J06 [38]) with a main chain root-mean-square deviation (RMSD) of 0.13 Å. No major structural changes of the protein backbone were evident. As for other structures of *m*AChE, the loop region including residues 258–264 could not be modelled from the acquired data. Moreover, the electron density around residue 495 is weak, resulting in a few outliers in the Ramachandran plot. The electron density maps convincingly define the binding site of C5685, with the substituted phenyl-ring system stacked between the phenol-ring of Tyr341 and the indole-ring of Trp286 (Figure 4A and 4B). The electron density map suggests that the *N*-methyl groups are in a plane with the aromatic system whereas the two oxygen atoms of the nitro group are out of the plane, thus presumably preventing a clash with the *N*-methyl groups. This allows a 3.0 Å hydrogen bond between the two nitro oxygens of C5685 and the main chain nitrogen of Phe295 (Figure 4B). The 2-methoxy group is found in the vicinity of the side chain of Asp74, forming a 2.9 Å intra-molecular hydrogen bond with the amide nitrogen of the linker. Moreover, the phenolic oxygen of Tyr124 is found at distances of 3.2 Å from the methoxy oxygen presumably allowing formation of a hydrogen bond between these groups (Figure 4B). In this particular structure, the electron density map of the Tyr337 side chain is disordered and was refined for two conformers (Figure 4). Only one of the conformers was present in the five other structures (corresponding to ATYR in the pdb-file). The *N*-ethylpyrrolidine moiety of C5685, containing the chiral carbon, is directed towards the CAS of *m*AChE but it could not be unambiguously modelled in the electron density map and was therefore omitted from the final structure. However, residual positive density features in the vicinity of the indole ring of Trp86 likely accounts for the unmodelled parts of the molecule (Figure 4A). Moreover, as often observed in structures of *m*AChE, a strong residual positive density feature is found close to Ser203Oγ.

**Table 2:** *IC_50_* values of the hits for which crystal structures were successfully determined.

Compound	*IC_50_* 1 (µM)
C7653	0.20±0.05
C7643	0.54±0.03
C5685	1.3±0.1
C6905	2.0±0.1
C7491	21.4±1.7
C5231	36.0±6.3

1 Mean ± standard deviations of 2–5 determinations.

**Table 3:** Interactions patterns observed in the determined complexes between the ligands and the corresponding residues in *m*AChE.^1^

	Inhibitor					
Residue	C5231	C5685	C6905	C7491	C7643	C7653
Trp286	x	x	x	x	x	x
Tyr341	x	x	x	x	x	x
Phe295	x	x	x	x	x	x^2^
Tyr124	-	x	x	-	x	-
Tyr337	-	-	x	x	-	x
Trp86	-	-3	-	-	x	x

1 x indicates presence of an interaction defined by close contacts (pair-wise heavy atom distance ≤3.5 Å).

2 The interaction is mediated by a water molecule.

3 Residual positive density features were found within the cut off distance.

**Table 4:** Data collection parameters and refinement statistics.

Data collection	C5685•*m*AChE
PDB entry code	4A23
Space group	P2_1_2_1_2_1_
Unit cell dimensions (Å)	78.9×111.8×227.0
Resolution range (Å)	19.64–2.40 (2.4–2.53)
Total number of reflections	585242 (85350)
Unique reflections	79036 (11407)
Completeness (%)	99.6 (99.8)
Multiplicity	7.4 (7.5)
*R*_merge_ 1	0.053 (0.358)
Mean(I)/sd(I)	25.8 (7.5)
**Refinement**	
*R*-factor^2^/*R*_free_ 3 (%)	17.5/20.9
*B*-factor^4^ (Å^2^)	48.24
Number of water molecules	871
**RMSD from ideal values**	
Bond lengths (Å)	0.008
Bond angle (°)	1.135
**Ramachandran plot %/no. of residues**	
Most favoured regions	1030 (97.2%)
Allowed regions	26 (2.5%)
Residues in disallowed regions	4 (0.4%)^5^

1 R*_merge_* = (∑|*I*−<*I*>|)/∑*I*, where *I* is the observed intensity and <*I*> is the average intensity obtained after multiple observations of symmetry related reflections.

2 R-factor = (∑∥*F_o_*|−|*F_c_*∥)/∑*F_o_*, where *F_o_* are observed and *F_c_* calculated structure factors.

3 R*_free_* uses 2% randomly chosen reflections defined in Brunger [77].

4 *B*-factor is the mean factor for protein main chain A/B.

5 Corresponds to Ala 342, Ala542, Lys496 and Ser497of the B monomer.

### Chemical space of acetylcholinesterase inhibitors

**Figure 5:**
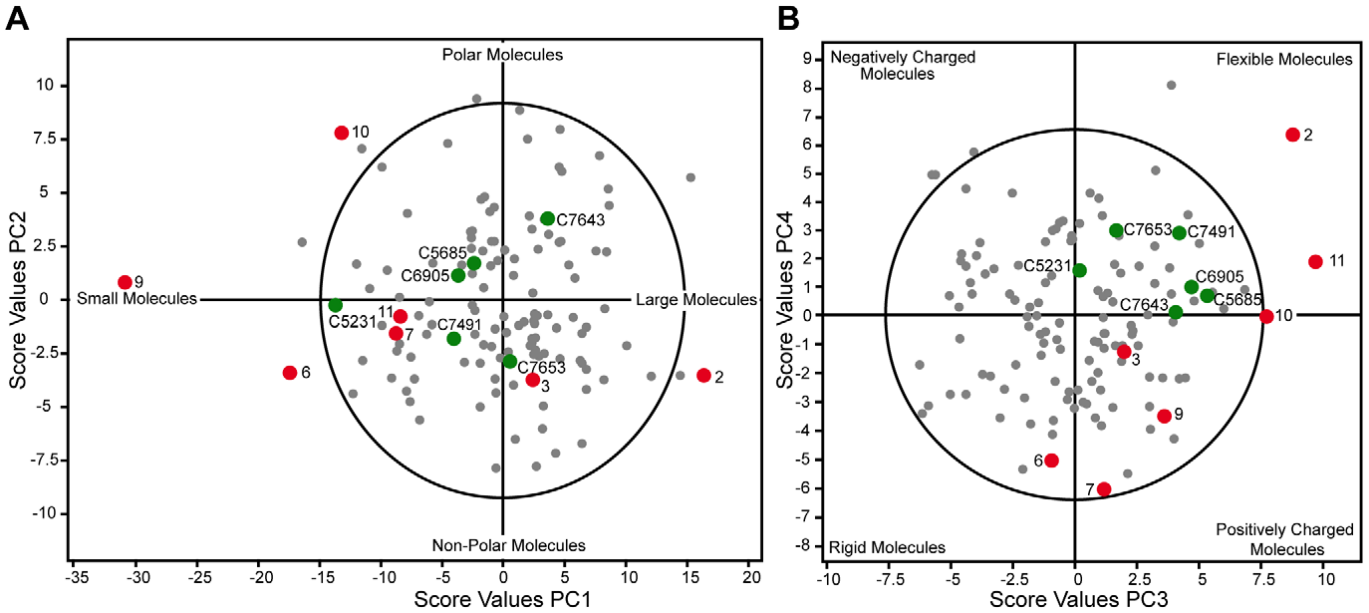
The chemical space of the identified AChE inhibitors. The chemical space was established by PCA of the physicochemical properties of the 124 hits (grey dots) that were identified in the HTS. The first and second PCs describe the size and polarity of the molecules (A), while the third and forth PCs illustrate the molecule's distribution related to charge and flexibility (B). The hits for which crystal structures were successfully determined are shown in green and the known AChE inhibitors that were projected into the chemical space are shown in red (**2**, **3**, **6**, **7**, and **9**–**11**; see Figure 1 for chemical structures). Hotelling's T2 (95%) is visualized as circled solid line.

The AChE inhibitors discovered in the screening campaign are chemically diverse, with molecular weights ranging between 234 a.u. and 596 a.u., logP (o/w) values between −1.16 and 8.14, and 0 to 12 rotatable bonds. The broad chemical diversity is further illustrated by a PCA of the structural and physicochemical features of the hits. Five principal components (PCs) proved significant: PCs 1 and 2 related mainly to size and hydrophobicity, respectively; PCs 3 and 4 related to flexibility *and* charge (positive, neutral or negative); and PC5 related to electronic properties associated with halogens and aromatic elements (see File S1). The 124 hits distributed evenly in the chemical space formed by these five PCs (Figure 5 and File S1). The 30 selected hits with determined *IC_50_* values (Set 1) confirmed that AChE inhibitors populated the established chemical space as no false positives were detected. No correlations were detected between the calculated structural and physicochemical features of the compounds and their inhibitory effect.

The hits for which crystal structures were successfully determined only covered part of the chemical space although the 36 hits that were subjected to complex-formation trials completely spanned the chemical space (Figure 5 and File S1). The ligands of the determined complexes showed a moderate span in size, hydrophobicity and electronic properties (i.e., PC1, PC2, and PC5) whereas they were relatively more flexible and lacked negative charges and thus did not spread completely in PC3 and PC4.

A selection of previously identified AChE inhibitors and reactivators (**2**, **3**, **6**, **7** and the scaffolds of **9**–**11** without the oxime functionality) were projected into the established chemical space. The PCA clearly shows that our discovered hits occupy a different and significantly larger chemical space (PC1 vs. PC2, and PC3 vs. PC4, Figure 5 and File S1).

### Molecular docking of acetylcholinesterase inhibitors

We set out to develop a general docking protocol for predicting binding modes of AChE inhibitors, which could be used for structure-based design in drug discovery projects. The ligands of the determined protein-ligand complexes (Figure 3) were docked to the protein conformation observed in the C5685•*m*AChE protein crystal structure, where the conformation of Tyr337 with the highest apparent occupancy was represented. In addition, 12 water molecules were explicitly included during the docking simulations, selected based on analysis of conserved water molecules in the binding site of *m*AChE.

The ability of three docking software packages (FRED [39], Glide [40] and GOLD [41]) to regenerate the bioactive conformations of the ligands was evaluated, deeming a docking pose with an RMSD value less than 2.0 Å compared to the pose found in the crystal structure to be acceptable. The investigated packages differ in their treatment of ligand and protein flexibility as well as how the docking poses are generated in the binding site of the protein (for details, see references for the respective packages). When default parameter settings were used, all three packages generated poor pose predictions (Table 5). However, Glide had the best success rate, generating acceptable poses for two of the seven ligands. It should be noted that adjustments of the dimensions used to define the binding site were made to allow placement of the ligands in any part of the active site gorge during the docking simulations.

**Table 5:** RMSD values of poses generated by molecular docking simulations of AChE inhibitors using default parameter settings.

	RMSD (Å)^1^						
Docking software	C5231	C5685 (*R*)	C5685 (*S*)	C6905	C7491	C7643	C7653
FRED	6.83	4.14	3.89	3.67	3.07	2.44	10.49
Glide	6.79	**1.14**	7.83	2.63	3.69	**1.13**	2.04
GOLD^2^	8.53	8.16	2.50	4.89	3.87	2.77	**1.77**

1 The RMSD values of the acceptable poses (less than 2.0 Å) are indicated in bold.

2 The RMSD value (Å) of the highest ranked pose by GoldScore is reported from the GOLD docking.

**Figure 6:**
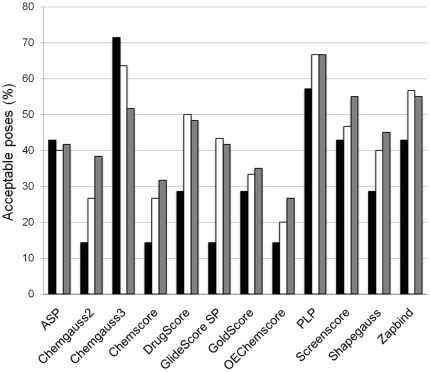
The proportion of acceptable poses highly ranked by each of the scoring functions. The percentage of poses with RMSD values less than 2.0 Å compared to the pose found in the crystal structure among the highest ranked (black), among the top five (white) and among the top ten poses (grey) is presented. Since there were few acceptable poses of C5231, the ligand was only considered in the calculation of the percentage among the highest ranked.

To improve the performance of the docking software, modified parameter settings were applied in Glide, where the number of poses included in the post-docking force-field minimization and the number of poses to output was increased. Ensembles of poses were thereby generated, including acceptable poses for all ligands. However, the acceptable poses were not found among those top-ranked by the scoring function used for docking in standard precision mode in Glide, GlideScore SP (Table 6). All poses were therefore re-scored using available scoring functions in GOLD (ASP and GoldScore), and FRED (Chemgauss2, Chemgauss3, Chemscore, OEChemscore, PLP, Screenscore, Shapegauss and Zapbind) as well as with DrugScore. Chemgauss3 proved to be the best performing scoring function, top-ranking acceptable poses for five of the seven ligands (Table 6). When the number of total acceptable poses among the five and ten top ranked by the different scoring functions were examined, PLP was the superior scoring function resulting in 65% of the poses with RMSD value less than 2.0 Å compared to the pose found in the crystal structure (Figure 6). The worst performing scoring functions were Chemgauss2, Chemscore and OEChemscore top-ranking acceptable poses for only one of the six ligands and less than 30% acceptable poses ranked in the top five (Table 6 and Figure 6). The lowest RMSD-values among the five and ten highest ranked docking poses by the twelve scoring functions can be viewed in File S1. Based on our results, the choice of scoring function will depend on whether several docking poses will be extracted for post-processing and re-scoring. If only the top ranked pose is to be extracted, Chemgauss3 appears to be the best choice, while PLP seems to be a better option if further processing of multiple poses is intended.

**Table 6:** RMSD values of the highest ranked docking poses after re-scoring of poses generated using the modified parameter settings in Glide.^1^

	RMSD (Å)^2^						
Scoring function	C5231	C5685 (*R*)	C5685 (*S*)	C6905	C7491	C7643	C7653
ASP^a^	7.02	3.75	3.75	**1.12**	3.13	**1.54**	**1.35**
Chemgauss2^b^	6.92	3.41	**1.35**	3.17	4.59	11.24	2.04
Chemgauss3^b^	6.84	**1.14**	**1.02**	**1.40**	3.02	**0.83**	**1.46**
Chemscore^b^	6.66	6.18	6.07	**1.51**	8.55	2.18	2.02
DrugScore	6.89	8.26	**1.35**	**1.08**	5.02	2.20	2.68
GlideScore SP^c^	6.79	**1.12**	3.97	3.23	3.02	2.20	2.04
GoldScore^a^	7.05	8.39	7.83	**0.81**	8.90	**1.94**	2.05
OEChemscore^b^	8.73	6.20	6.07	**1.17**	5.02	11.68	2.05
PLP^b^	6.84	**1.00**	**1.02**	**1.16**	9.09	**1.19**	2.05
Screenscore^b^	7.02	6.16	6.94	**1.51**	2.77	**1.19**	**1.88**
Shapegauss^b^	8.23	8.26	2.95	**1.51**	4.41	**0.83**	2.32
Zapbind^b^	6.93	4.03	2.69	2.41	**1.46**	**0.90**	**1.20**

1 Scoring functions are available in:

a GOLD,

b FRED and

c Glide.

2 The RMSD values of the acceptable poses (less than 2.0 Å) are indicated in bold.

**Figure 7:**
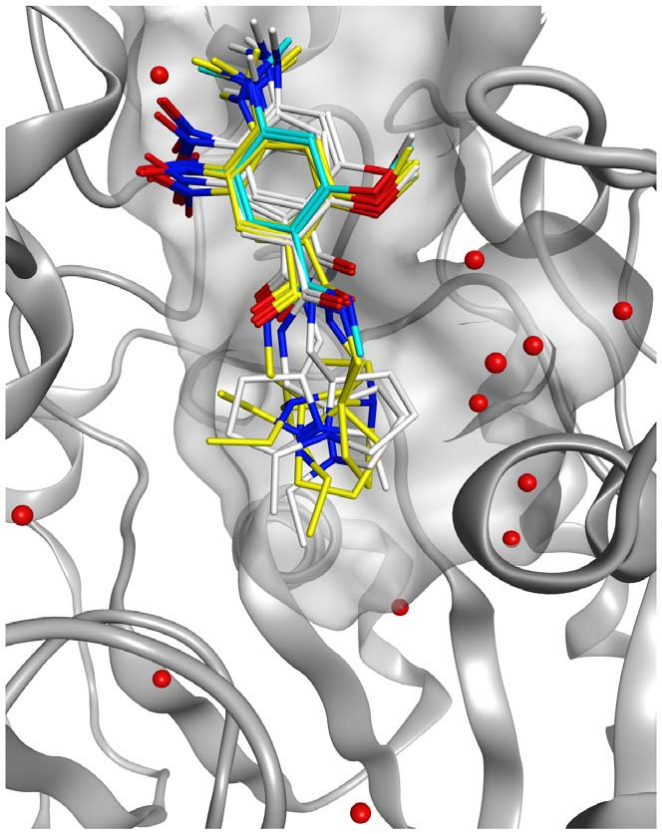
Predicted binding poses of both C5685 enantiomers by molecular docking simulations. Poses of C5685 (*R*) and C5685 (*S*) that were generated using the modified parameter settings in Glide are shown with carbons colored white and yellow, respectively. The part of C5685 as modeled in the X-ray crystal structure is shown with carbons colored in cyan, and the 12 conserved water molecules that were included in the docking simulations are indicated in red.

The generated poses for the C5685 enantiomers were evaluated to investigate possible binding modes of the part of the molecule that was not modeled in the X-ray crystal structure (i.e., the *N*-ethylpyrrolidine moiety). Acceptable poses were obtained for both C5685 (*R*) and C5685 (*S*), and manual inspection of the docking poses revealed that a number of conformations of the *N*-ethylpyrrolidine substructure had been generated. To investigate if the binding modes proposed by Glide are structurally relevant, the local strain energies of the ligand conformations were calculated. For each enantiomer, three poses with low local strain energy were selected as representative poses (Figure 7). The local strain energies of these poses are of the same order of magnitude as for the ligand conformations of the five other included protein-ligand complexes.

## Discussion

### AChE inhibitors as chemical leads for drug discovery

The primary goals of the present study were to identify novel AChE ligands using HTS of a chemical library and to evaluate if a combination of crystallography and attentive molecular docking can be used to design drugs targeting AChE. While numerous inhibitors and their crystal structures have been reported previously, the 124 hits from the screening campaign reported herein were identified from a large, unbiased library of 17 500 compounds. The identified hits encompass large chemical diversity (including for instance small, large, polar, non-polar, rigid, flexible, acidic and basic compounds) and they are structurally different from the AChE inhibitors derived from natural products as well as the known reactivators. Current drugs targeting AChE, *e.g*., to treat symptoms of Alzheimer's disease, are associated with adverse effects, and/or low efficacy [14], warranting searches for new chemical skeletons that could have different pharmacodynamic and pharmacokinetic profiles. Our hits could serve as chemical leads in such drug discovery programs. Furthermore, the discovered hits have different chemical skeletons from the currently used nerve agent antidotes, and could be used in the development of antidotes with increased blood-brain barrier permeability and a broader reactivation spectrum.

One of the challenges associated with this work was to obtain high-resolution structural data for a sufficient number of hits to obtain information regarding binding modes that could provide starting points for docking-assisted structure-based design. Crystallographic study of AChE complexes often involves optimization of the ligand occupancy through spectroscopic techniques or the collection of several datasets under varying conditions [20], [38], [42]. Optimization of the ligand occupancy is advantageous and feasible in investigations of a limited number of complexes. However, the number of hits we identified prompted a different approach, relying exclusively on a standardized protocol.

Starting with a collection of 36 hits, over 200 soaking experiments were performed, resulting in the collection of 35 datasets and seven finalized structures, corresponding to an overall success rate of 19%. The structural analysis of the ligands shows that their non-covalent interactions with AChE have several common features. All crystal structures show extensive interactions between the ligand and the PAS region of AChE. One ligand is PAS–specific, whereas the remaining structures have moieties that extend towards the catalytic site and the indole ring of Trp86. Of the compounds for which AChE-ligand complexes have been previously determined, donepezil **3** is chemically most similar to our structurally determined AChE inhibitors (cf. **3** and green markers in Figure 5). Comparison of the crystal structures C5685•*m*AChE and **3•** *Tc*AChE (pdb entry code: 1EVE [21]) shows that the overall protein structures are similar and that the ligands occupy the same portion of the binding site. The PAS-binding parts show similar molecular interaction patterns, including the direct or water-mediated hydrogen bond between the ligands and the main chain nitrogen of Phe295. The main differences in structure and molecular interaction patterns can be attributed to the side-chain differences (Tyr337 in *m*AChE and Phe330 in *Tc*AChE). Molecular docking simulations were used to investigate possible binding modes of the *N*-ethylpyrrolidine moiety of C5685. Several plausible binding modes were detected and no distinction could be made between the two enantiomers in this respect; low RMDS-poses that represent structurally relevant binding modes were generated for both C5685 (*R*) and C5685 (*S*).

Reproducing the bioactive conformations of six ligands (determined by crystallography) using molecular docking proved to be difficult. Applying the default parameter settings, no docking program could reproduce the experimentally determined binding modes in a satisfactory manner. One challenge appears to be the large binding site with similar physicochemical features of PAS and CAS, leading to inaccurately predicted binding modes in which the ligands are rotated 180 degrees. By modifying the docking parameters then re-scoring the output poses using 12 scoring functions, acceptable results were obtained. The results from the re-scoring showed that the latest version of Chemgauss (*i.e.* Chemgauss3), a Gaussian-based empirical model that include a solvatisation term, gave good results together with PLP. The scoring functions that are based on the Chemscore scoring functions (Chemscore, OEChemscore and Glidescore) seem to give less reliable results. It should be noted that the general applicability of these results needs to be investigated. Previous studies involving re-docking of natural products with known bioactive conformations have shown that the type of ligand, the protein conformation and the presence of water are important factors [24]–[26]. In general, we believe that molecular docking of ligands to AChE for predicting the binding modes is very challenging, leading to large uncertainties in the results, and thus must be accompanied by X-ray crystallography to experimentally determine the bioactive conformation of several ligands representing the chemical skeleton of interest.

### Future design of nerve agent antidotes

It is important to emphasize that the hits identified and characterized in the present study were selected from a library composed of drug-like molecules. In contrast to reactivators (*i.e.* nerve agent antidotes), they lack reactive nucleophilic moieties, and are therefore unable to restore the function of AChE inhibited by nerve agents (OP-AChE). Thus, the criteria, experimental data and techniques required to design reactivating antidotes using these hits need to be identified and developed. The efficacy of a reactivator is described by its bimolecular reactivation constant (*k_r2_*), which depends on both the affinity (dissociation constant, *K_D_*) between the antidote and the inhibited enzyme, and the rate of the chemical reaction (*k_r_*). The determination of *k_r2_* has a limited utility in rational design of new reactivators since only molecules that contain a nucleophilic moiety *and* show reactivation activity will result in *k_r2_* values that can guide further molecular development. Thus, we are currently developing activity-independent methods based on time correlated single photon counting spectroscopy in order to determine the binding affinity of candidate molecules to inhibited AChE.

Based on our hits and determined crystal structures (Figure 3), we have identified chemical entities that interact with the PAS, the active site gorge, or the CAS. Our approach will now be to use these chemical entities in statistical molecular design [43] to construct small sets of compounds formed by combinations of PAS-, gorge-, and CAS-specific fragments that can be submitted for synthesis and explored for binding affinity of OP-AChE to identify suitable chemical leads for antidotes. We believe that a key property of the nucleophile is an intrinsic mobility that allows a conversion to the transition state for reactivation while preventing entrapment in an unproductive conformation. The design of the positioning of the nucleophile on the chemical leads will be assisted by molecular docking based on the proposed protocol followed by molecular dynamic simulations.

### Conclusions

In presented study, *in vitro* screening of 17 500 drug-like molecules resulted in 124 hits that inhibited the enzymatic activity of AChE by more than 70%. Re-testing at several ligand concentrations confirmed their activity, with *IC_50_* values of 0.20–82 µM. The discovered hits displayed a wide chemical diversity and were structurally different from known inhibitors and reactivators. In an extensive crystallization effort we successfully determined the 3D structures of seven of these ligands in complex with *m*AChE. All structures showed extensive interactions between the ligand and amino acids in PAS and five of the structures had additional interactions with the active site gorge and CAS. Reproduction of the bioactive conformations of the ligands using molecular docking by Glide required modification of the molecular docking protocol. The multiple crystal structures provided a robust test of the scoring functions used for re-scoring, for which Chemgauss3 and PLP, proved to be superior for this data set. The results show that structure-based design of AChE inhibitors and reactivators using molecular docking simulations needs to be assisted by crystallography to obtain reliable results. The C5685•*m*AChE crystal structure represents the general mode of binding in PAS and the developed docking protocol was used to predict the binding modes of the C5685 enantiomers at CAS, which were not modeled in the X-ray crystal structure. According to the molecular docking simulations both C5685 (*R*) and C5685 (*S*) will bind to AChE as acceptable binding poses were obtained for both.

The design of AChE inhibitors and nerve agent antidotes as outlined herein is an experimentally challenging drug discovery project for which mechanistic insights together with structural, biochemical and biophysical techniques are required as foundations for (and to validate) computational techniques such as molecular docking and dynamic simulations. We believe that chemical modifications of our discovered inhibitors, biochemical and biophysical characterization, crystallography and computational chemistry provide a route to novel AChE inhibitors and potentially also to reactivators.

## Materials and Methods

### High throughput screening

The screening was performed at the Umeå Small Molecule Screening Facility currently incorporated in the screening platform of the Laboratories for Chemical Biology, Umeå (LCBU) [44]. Recombinant *h*AChE was expressed and purified according to previously described methods [45], [46]. The enzymatic activity was measured using the Ellman assay [47] adapted to a 96-well format. The final assay volume was 200 µL and all measurements were performed in 0.2 mM 5,5′-Dithiobis(2-nitrobenzoic acid) and 1 mM acethylthiocholine iodide in 0.1 M phosphate buffer pH 8.0. The chemical collection comprising 17 500 unique compounds accessed by LCBU has been purchased from ChemBridge (San Diego, CA) [44]. Stock solutions were prepared in DMSO and transferred to the assay plate using a Biomek NC robotic system (BeckanCoulter). The enzymatic reaction was monitored for 120 seconds using an Infinite M200 plate-reader (Tekan) at a ligand concentration of 50 µM and temperature of 20°C. Each plate contained 80 samples, 14 positive controls and two negative controls. The average slope (typically 0.48±0.1 dA/min) obtained from assays with 14 positive controls was set as 100% activity. Substances reducing the activity by at least 70% were scored as hits. *IC_50_* values were determined using a similar approach, starting with freshly prepared solution of the inhibitor. For each inhibitor, the enzymatic activity was determined at eight concentrations selected to produce a dose-response curve that was subsequently analyzed using Prism [48].

### Selection of Sets 1 and 2

Set 1 includes compounds that were identified as hits in the HTS, for each of which a full dose response curve was obtained to verify its inhibitory activity (and thus detect potentially false positives). This set was selected manually from the chemical space spanned by the hits (i.e. the five significant PCs; Figure 5 and File S1) in two rounds (20 plus 10 compounds). The selection was made to achieve an even distribution in the chemical space without including compounds with extreme values for any of the significant PCs. Set 2 includes compounds that showed *no* activity in the HTS but had similar structural and physiochemical features to the hits and was used to investigate the degree of false negatives. Set 2 was selected in a similar fashion to Set 1, based on predicted scores resulting from projection of compounds with 0±5% activity in the HTS into the chemical space of the AChE inhibitors. Set 2 was also selected in two rounds (20 plus 10 compounds). All of the included inactives occupied the same chemical space as the AChE inhibitors, that is, they exhibited similar chemical features as the hits.

### Generation, collection and refinement of crystal structures

*m*AChE was crystallized as previously described [45]. Grains of the ligand were added to a soaking solution consisting of 30% (v/v) polyethylene glycol 750 monomethylether, 100 mM HEPES pH 7.0 until saturation was reached and a precipitate was formed. After approximately 15 minutes the precipitate was removed by centrifugation and the soaking solution was added in four portions of 2 µL to a crystal of *m*AChE. The soaking was performed during a time-frame of five minutes and the crystal was incubated for an additional five minutes prior to flash-freezing in liquid nitrogen. X-ray diffraction data were collected at the MAX-lab synchrotron (Lund, Sweden), beam lines I911-2 and I911-3, equipped with MAR Research CCD detectors. The images (at least 140) were collected using an oscillation angle of 1.0° per exposure. The intensity data were indexed and integrated using XDS [49] and scaled using Scala [50]. The C5685•*m*AChE structure was determined using rigid-body refinement starting with a modified *apo* structure of *m*AChE (PDB entry code: 1J06 [38]). Further crystallographic refinement was performed using the Phenix software suite [51]. The presence of ligands in the AChE binding site of the AChE crystals was estimated based on the initial 2|*F_o_*|−|*F_c_*| and |*F_o_*|−|*F_c_*| omit maps, and only protein-ligand complexes where ligand occupancies were detected were subjected to further refinement. Several rounds of refinement were performed, alternating with manual rebuilding of the model after visualizing the 2|*F_o_*|−|*F_c_*| and |*F_o_*|−|*F_c_*| electron density maps using COOT [52]. The quality of the final model was evaluated using PROCHECK, WHATCHECK and RAMPAGE [53]–[55] and the figures were constructed using PyMol [56].

### Chemical space of acetylcholinesterase inhibitors

The structural and physicochemical features of the 17 500 compounds in the chemical library were described by 2D molecular descriptors using MOE [57]. A complete list of descriptors is shown in File S1. The properties of the hits from the HTS were used to extract the main principal properties of the AChE inhibitors, and hence form the chemical space they spanned, using PCA. PCA is an unsupervised projection method in which systematic variation in a data set is extracted into a few variables; Principal Components or PCs [58]. The PCs are linear combinations of the original variables and are uncorrelated to each other, as described by Equation 1.
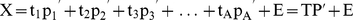
(1)where **X** is the original data matrix (here the chemical features' of the compounds), *A* is the total number of extracted PCs, and **E** is the residual matrix. The new latent variables, the **t** scores, show how the compounds relate to each other, while the **p** loadings reveal the importance of the original chemical features for the patterns seen in the scores. The number of significant components was determined according to a Scree-plot (eigenvalues vs. PCs, see File S1). The data were mean-centered and scaled to unit-variance prior to PCA. The analysis was performed using SIMCA [59] and Evince [60]. Model statistics are found in File S1.

### Projection of known AChE ligands

To monitor and assess the novelty of the chemical features of the discovered hits, the positions of **2**, **3**, **6**, **7**, and **9**–**11** were projected into the chemical space established by the AChE inhibitors. The comparison with **9**–**11** was based on the scaffolds of the reactivators, and the oxime functionality was therefore removed prior to calculating the molecular descriptors.

### Molecular docking

The atomic coordinates of six of the protein-ligand complexes determined by X-ray crystallography (C5231, C5685, C6905, C7491, C7643 and C7653) were considered in the docking study. All dockings were performed to one representative protein crystal structure (C5685•*m*AChE) as the objective was to generate a general docking protocol that could be used to predict the binding poses of new ligands. Default settings were used in the computations unless otherwise stated.

#### Ligand and protein preparation

The 3D atomic coordinates of the ligands used as input in the docking simulations were rebuilt from SMILES in MOE [57] using the MMFF94x force field. Both of the enantiomers of C5685 were included in the docking attempts, resulting in a total of seven ligands. The ligand protonation states were set manually, assigning amines positive charges while anilines were considered neutral, rendering six out of seven ligands positively charged. All ligands were docked to the C5685•*m*AChE protein crystal structure (refined to a resolution of 2.4 Å), where the most populated conformation of Tyr337 was selected (ATYR337) with an apparent occupancy of 0.57. This conformation was selected since it is similar to the conformations observed in the remaining protein-ligand complexes included in the study. Twelve conserved hydration sites were identified in *m*AChE by manual inspection of water molecules present in 18 crystal structures available from the RCSB protein data bank [61] (pdb entry codes: 1KU6, 1N5M, 1Q84, 2GYU, 2GYV, 2GYW, 2H9Y, 2HA0, 2HA2, 2HA3, 2HA4, 2JEZ, 2JF0, 2JGE, 2JGL, 2WHR, 3DL4 and 3DL7) and the structures determined herein. The results from this analysis were transferred to the C5685•*m*AChE protein crystal structure where all water molecules were removed from the coordinate file except for W3, W9, W15, W46, W84, W85, W127, W145, W176, W229, W339 and W729. The *protein preparation wizard* implemented in Maestro [62] was used to prepare an all-atom protein model and to optimize the hydrogen bond network. Only the A chain of the protein, including the specified water molecules, was retained after processing. The His447 residue was manually set to a mono-protonated state. Residues with unmodelled side chain atoms were predicted using Prime [63].

*Docking in FRED* [39]. The receptor file was prepared in FRED receptor [64] in which the binding site was defined by a box with a volume of 5580 Å^3^, with inner and outer contour levels of 333 Å^3^ and 1453 Å^3^, respectively. Conformational ensembles of the ligands used as input were generated in OMEGA [65], and docking was performed using default parameter settings.

#### Docking in Glide [40], [66]

The receptor grid was prepared from the command line using default settings except for the dimensions of the *ligand diameter midpoint box* and the *enclosing box*, for which the side lengths were increased to 20 Å and 35 Å, respectively. The binding site was defined from the *x*-, *y*-, *z*-coordinates 31.30, 22.70, and 9.80, respectively. Docking was performed in standard precision mode using either default settings or modified parameter settings (increasing the number of poses to include during the post-docking minimization and the number of output poses to 1000 and 100 per ligand, respectively).

#### Docking in GOLD [41], [67], [68]

The binding site was defined as a radius of 15 Å from the hydroxyl oxygen of Tyr337. The 12 water molecules included in the coordinate file were active and allowed to spin during the docking simulation. Docking was performed using default parameter settings. The pose ranked highest by GoldScore was considered from the docking.

#### Evaluation of output poses

RMSD values (Å) of the heavy atoms of the generated poses compared to the crystallographically determined ligand conformations were calculated in MOE [57], [69]. RMSD values for the two C5685 enantiomers were based on the part of the ligand that was modeled and included in the final C5685•*m*AChE structure (i.e. excluding the atoms of the *N*-ethylpyrrolidine moiety, see File S1). To enable RMSD calculations for all included ligands, the atomic coordinates of the protein-ligand complexes were superposed to the C5685•*m*AChE protein structure using the *protein superpose* panel in MOE [57]. A successfully regenerated binding mode was defined as having an RMSD value less than 2.0 Å. The poses that were generated using the modified parameter settings in Glide were scored using twelve scoring functions available in Glide [40], [66], GOLD [41], [67], [68] and FRED [39] as well as with Drugscore (version 1.3). The scoring functions include force field-based (GoldScore), empirical (GlideScore SP, Chemscore [70], OEChemscore, PLP [71], Screenscore [72]), Gaussian-based empirical (Chemgauss2, Chemgauss3, Shapegauss [73], Zapbind [74]) and knowledge-based (DrugScore [75] and ASP [76]). Prior to re-scoring using Zapbind, the poses generated by Glide were refined using the Merck Molecular Mechanics Force Field (MMFF) according to recommendations from OpenEye. The RMSD values of the poses ranked by Zapbind are reported for the refined poses.

#### Calculation of local strain energies

Local strain energies were calculated in MOE [57] for all poses of C5685 (*R*) and C5685 (*S*) generated using the modified parameter settings from Glide with RMSD values less than 2.0 Å. A constrained relaxation of the docking poses was performed to remove incompatibilities between the force field used during the docking (OPLS_2001) and the force field used for the calculations of the potential energies (MMFF94s), during which all heavy atoms were tethered using a weight of 10. The docking poses were thereafter fully energy-minimized to the closest local minima. The local strain energy was calculated as the difference in potential energy between the relaxed ligand conformation and the fully energy-minimized conformation. The same analysis was performed for the ligand conformations observed in the other determined protein-ligand complexes that were included in the docking study.

## Supporting Information

File S1 **Molecular descriptors used in the PCA of small organic molecules. Re-testing of the representative set of compounds that were identified as non-binders to AChE in the HTS.** Model statistics for the significant principal components. The lowest RMSD values among the five highest ranked docking poses after re-scoring. The lowest RMSD values among the ten highest ranked docking poses after re-scoring. Scree-plot (eigenvalue vs. principal component) of eight components. Score plot (PC5). Loading plots (PC1–PC5). Distribution of the hits subjected to crystallization trials in the score plots (PC1–PC4). Overlay of C5685 docking poses with the X-ray crystal ligand.
(DOC)
