# Supplementary material for: Targeting Acetylcholinesterase: Identification of Chemical Leads by High Throughput Screening, Structure Determination and Molecular Modeling
Source: PLoS One. 2011 Nov 30;6(11):e26039. doi: 10.1371/journal.pone.0026039 (PMC3227566; doi:10.1371/journal.pone.0026039)
Supplement: File S1 — Molecular descriptors used in the PCA of small organic molecules. Re-testing of the representative set of compounds that were identified as non-binders to AChE in the HTS. Model statistics for the significant principal components. The lowest RMSD values among the five highest ranked docking poses after re-scoring. The lowest RMSD values among the ten highest ranked docking poses after re-scoring. Scree-plot (eigenvalue vs. principal component) of eight components. Score plot (PC5). Loading plots (PC1–PC5). Distribution of the hits subjected to crystallization trials in the score plots (PC1–PC4). Overlay of C5685 docking poses with the X-ray crystal ligand. (DOC) [file pone.0026039.s001.doc]

**SUPPORTING INFORMATION**

## Targeting acetylcholinesterase; Identification of Chemical Leads by High Throughput Screening, Structure Determination and Molecular Modeling

Lotta Berg^1^, C. David Andersson^1^, Elisabet Artursson^2^, Andreas Hörnberg^2^, Anna-Karin Tunemalm^2^, Anna Linusson^1*^and Fredrik Ekström^2*^

^1^Department of Chemistry, Umeå University, Umeå, Sweden

^2^Swedish Defence Research Agency, CBRN Defence and Security, Umeå, Sweden

*E-mail: fredrik.ekstrom@foi.se and anna.linusson@chem.umu.se,

**Table S1.** Molecular descriptors used in the PCA of small organic molecules.

**Table S2.** Re-testing of the representative set of compounds that were identified as non-binders to AChE in the HTS.

**Table S3.** Model statistics for the significant principal components.

**Table S4.** The lowest RMSD values among the five highest ranked docking poses after re-scoring.

**Table S5.** The lowest RMSD values among the ten highest ranked docking poses after re-scoring.

**Figure S1.** Scree-plot (eigenvalue vs. principal component) of eight components.

**Figure S2.** Score plot (PC5).

**Figure S3.** Loading plots (PC1-PC5).

**Figure S4.** Distribution of hits subjected to crystallization trials in the score plots (PC1-PC4).

**Figure S5.** Overlay of C5685 docking poses with the X-ray crystal ligand.

**Table S1.** Molecular descriptors*^a^* used in the PCA of small organic compounds.

| No. | Variable | No. | Variable | No. | Variable |
| --- | --- | --- | --- | --- | --- |
|  |  |  |  |  |  |
| 1 | **diameter** | 31 | **a_nO** | 60 | **Kier2** |
| 2 | **radius** | 32 | **a_nS** | 61 | **Kier3** |
| 3 | **VDistEq** | 33 | **b_heavy** | 62 | **KierA1** |
| 4 | **VDistMa** | 34 | **chi0** | 63 | **KierA2** |
| 5 | **weinerPath** | 35 | **chi0_C** | 64 | **KierA3** |
| 6 | **weinerPol** | 36 | **chi1** | 65 | **KierFlex** |
| 7 | **a_aro** | 37 | **chi1_C** | 66 | **logS** |
| 8 | **a_count** | 38 | **FCharge** | 67 | **apol** |
| 9 | **a_nH** | 39 | **VAdjEq** | 68 | **bpol** |
| 10 | **b_1rotN** | 40 | **VAdjMa** | 69 | **mr** |
| 11 | **b_1rotR** | 41 | **zagreb** | 70 | **a_acc** |
| 12 | **b_ar** | 42 | **balabanJ** | 71 | **a_acid** |
| 13 | **b_count** | 43 | **PEOE_PC+** | 72 | **a_base** |
| 14 | **b_double** | 44 | **PEOE_PC-** | 73 | **a_don** |
| 15 | **b_rotN** | 45 | **PEOE_RPC+** | 74 | **a_hyd** |
| 16 | **b_rotR** | 46 | **PEOE_RPC-** | 75 | **vsa_acc** |
| 17 | **b_single** | 47 | **PEOE_VSA_FHYD** | 76 | **vsa_acid** |
| 18 | **b_triple** | 48 | **PEOE_VSA_FNEG** | 77 | **vsa_base** |
| 19 | **chi0v** | 49 | **PEOE_VSA_FPNEG** | 78 | **vsa_don** |
| 20 | **chi0v_C** | 50 | **PEOE_VSA_FPOL** | 79 | **vsa_hyd** |
| 21 | **chi1v** | 51 | **PEOE_VSA_FPOS** | 80 | **vsa_other** |
| 22 | **chi1v_C** | 52 | **PEOE_VSA_FPPOS** | 81 | **vsa_pol** |
| 23 | **rings** | 53 | **PEOE_VSA_HYD** | 82 | **SlogP** |
| 24 | **Weight** | 54 | **PEOE_VSA_NEG** | 83 | **SMR** |
| 25 | **a_heavy** | 55 | **PEOE_VSA_PNEG** | 84 | **TPSA** |
| 26 | **a_nBr** | 56 | **PEOE_VSA_POL** | 85 | **density** |
| 27 | **a_nC** | 57 | **PEOE_VSA_POS** | 86 | **vdw_area** |
| 28 | **a_nCl** | 58 | **PEOE_VSA_PPOS** | 87 | **vdw_vol** |
| 29 | **a_nF** | 59 | **Kier1** | 88 | **logP(o/w)** |
| 30 | **a_nN** |  |  |  |  |

*^a^* Calculated by MOE (*The Molecular Operating Environment, version 2009.10, software available from Chemical Computing Group Inc., 1010 Sherbrooke Street West, Suite 910, Montreal, Canada H3A 2R7*) based on chemical structures derived from the “wash” option.

**Table S2.** Re-testing of the representative set of compounds that were identified as non-binders to AChE in the HTS.

|  | Set 2 | |
| --- | --- | --- |
| No. | Compound | Activity^1^ |
| 1 | I7187 | - |
| 2 | I6394 | - |
| 3 | I9014 | - |
| 4 | I7958 | - |
| 5 | I9007 | - |
| 6 | I5572 | - |
| 7 | I7463 | - |
| 8 | I9026 | - |
| 9 | I5428 | - |
| 10 | I7989 | - |
| 11 | I5269 | - |
| 12 | I7707 | - |
| 13 | I7827 | - |
| 14 | I5304 | - |
| 15 | I7957 | - |
| 16 | I5357 | - |
| 17 | I7959 | - |
| 18 | I7977 | - |
| 19 | I5262 | - |
| 20 | I5664 | -^2^ |
| 21 | I7740 | - |
| 22 | I7986 | - |
| 23 | I7966 | - |
| 24 | I7943 | - |
| 25 | I9041 | - |
| 26 | I7963 | - |
| 27 | I5431 | - |
| 28 | I9012 | - |
| 29 | I9010 | - |
| 30 | I5733 | - |

^1^ The threshold for positive activity was set to a 70 % reduction of the enzymatic activity.

^2^ The compound showed a 71 % reduction of the enzymatic activity and an *IC_50_* value of 48 μM.

**Table S3.** Model statistics for the significant principal components.

| **Principal component** | **R^2^** | **R^2^ (cumulative)** | **Eigenvalue** |
| --- | --- | --- | --- |
| 1 | 0.40 | 0.40 | 35.05 |
| 2 | 0.15 | 0.55 | 13.56 |
| 3 | 0.10 | 0.66 | 9.19 |
| 4 | 0.08 | 0.73 | 6.64 |
| 5 | 0.04 | 0.77 | 3.30 |

**Table S4. The lowest RMSD values of the five highest ranked docking poses according to re-scoring of the poses generated using the modified parameter settings in Glide.^1^**

|  | **RMSD (Å)^2^** | | | | | | |
| --- | --- | --- | --- | --- | --- | --- | --- |
| **Scoring function** | **C5231** | **C5685 (*R*)** | **C5685 (*S*)** | **C6905** | **C7491** | **C7643** | **C7653** |
| ASP^a^ | 6.54 | **1.03** | **1.94** | **0.99** | 2.77 | **1.00** | **1.35** |
| Chemgauss2^b^ | 6.79 | **1.14** | **1.05** | 2.50 | 3.13 | **1.13** | 2.04 |
| Chemgauss3^b^ | 6.79 | **0.97** | **0.60** | **1.35** | 3.02 | **0.81** | **1.40** |
| Chemscore^b^ | 6.66 | 5.99 | 5.87 | **1.01** | 8.48 | **0.81** | **1.35** |
| DrugScore | 6.06 | **0.97** | **0.94** | **0.91** | 4.08 | **1.07** | **1.92** |
| GlideScore SP^c^ | 6.66 | **1.00** | **1.02** | **0.99** | 2.77 | **1.07** | 2.04 |
| GoldScore^a^ | 6.79 | **1.14** | 6.18 | **0,81** | 4.28 | **0.84** | **1.88** |
| OEChemscore^b^ | 6.79 | **1.28** | 3.75 | **1.08** | 4.51 | **0.69** | **1.35** |
| PLP^b^ | 6.79 | **0.97** | **1.02** | **0.94** | 9.00 | **0.83** | **1.88** |
| Screenscore^b^ | 6.79 | 3.72 | 2.15 | **0.96** | 2.77 | **0.83** | **1.81** |
| Shapegauss^b^ | 7.02 | **1.17** | 2.46 | **0.89** | 4.08 | **0.81** | **1.46** |
| Zapbind^b^ | 3.56 | **0.81** | 2.69 | **1.34** | **1.41** | **0.90** | **1.20** |

^1^Scoring functions are available in: ^a^GOLD, ^b^FRED and ^c^Glide.

^2^The RMSD values of the acceptable poses (less than 2.0 Å) are indicated in bold

**Table S5.** The lowest RMSD values of the ten highest ranked docking poses according to re-scoring of the poses generated using the modified parameter settings in Glide.^1^

|  | **RMSD (Å)^2^** | | | | | | |
| --- | --- | --- | --- | --- | --- | --- | --- |
| **Scoring function** | **C5231** | **C5685 (*R*)** | **C5685 (*S*)** | **C6905** | **C7491** | **C7643** | **C7653** |
| ASP^a^ | 6.47 | **0.97** | **1.02** | **0.89** | 2.77 | **0.83** | **1.35** |
| Chemgauss2^b^ | 6.75 | **1.13** | **0.60** | **0.89** | 3.02 | 1.12 | **1.95** |
| Chemgauss3^b^ | 6.75 | **0.97** | **0.60** | **1.08** | 3.02 | **0.81** | **1.40** |
| Chemscore^b^ | **1.96** | 4.24 | **1.02** | **1.01** | 5.02 | **0.60** | **1.35** |
| DrugScore | 4.97 | **0.97** | **0.94** | **0.89** | 4.08 | **1.07** | **1.92** |
| GlideScore SP^c^ | 6.66 | **1.00** | **0.94** | **0.99** | 2.77 | **1.07** | **1.40** |
| GoldScore^a^ | 6.47 | **1.14** | **1.13** | **0.81** | **1.17** | **0.84** | **1.40** |
| OEChemscore^b^ | 6.79 | **1.28** | 3.75 | **1.04** | 4.28 | **0.69** | **1.35** |
| PLP^b^ | 6.66 | **0.97** | **0.48** | **0.94** | 4.28 | **0.83** | **1.35** |
| Screenscore^b^ | 6.66 | **0.97** | **1.02** | **0.96** | **0.71** | **0.83** | **1.46** |
| Shapegauss^b^ | 7.02 | **1.04** | **0.94** | **0.89** | 4.08 | **0.60** | **1.35** |
| Zapbind^b^ | 3.45 | **0.81** | 2.36 | **1.05** | **1.23** | **0.90** | **1.20** |

^1^Scoring functions are available in: ^a^GOLD, ^b^FRED and ^c^Glide.

^2^The RMSD values of the acceptable poses (less than 2.0 Å) are indicated in bold.


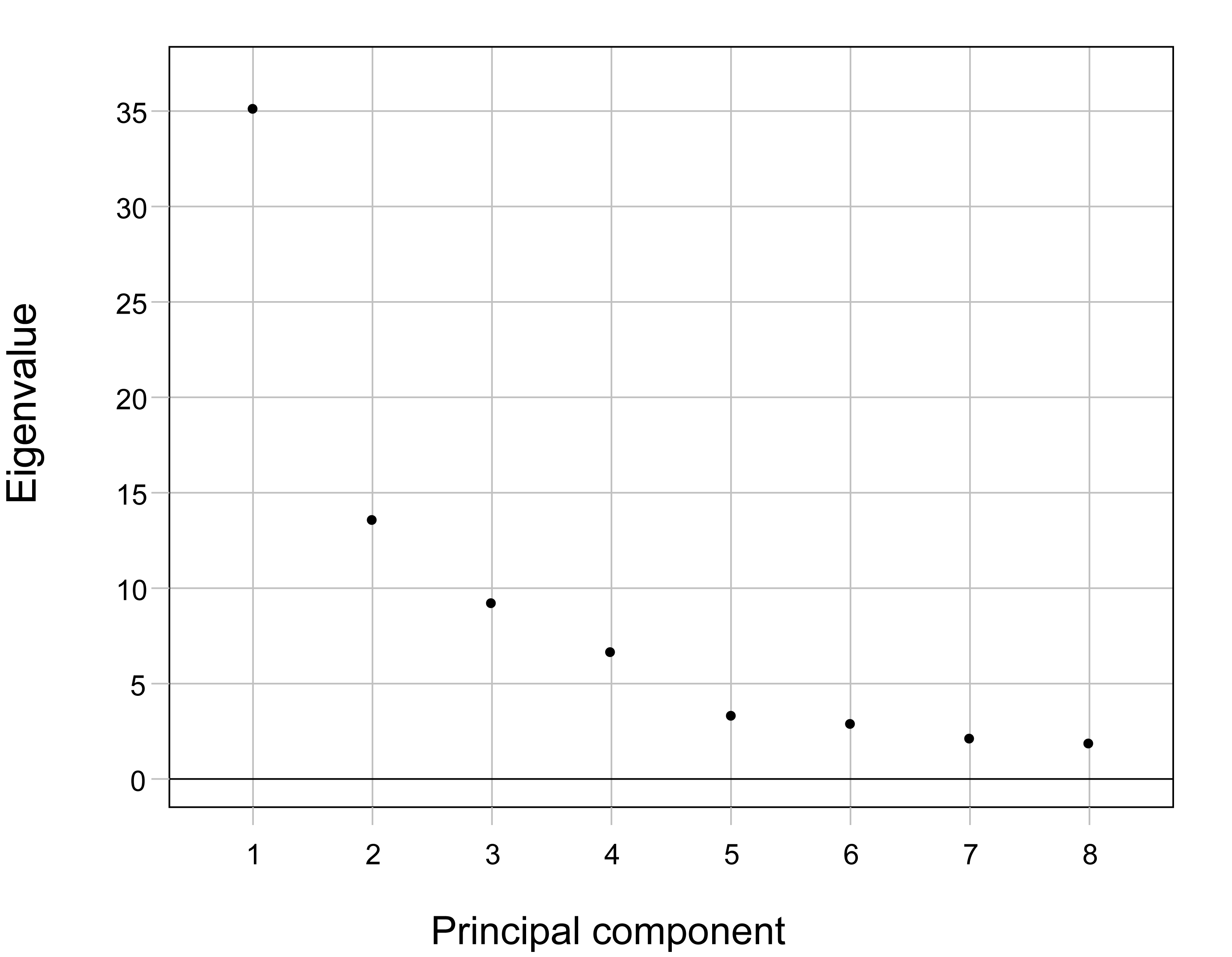


**Figure S1.** Scree-plot (eigenvalue vs. principal component) of eight components. Five components were regarded as significant.


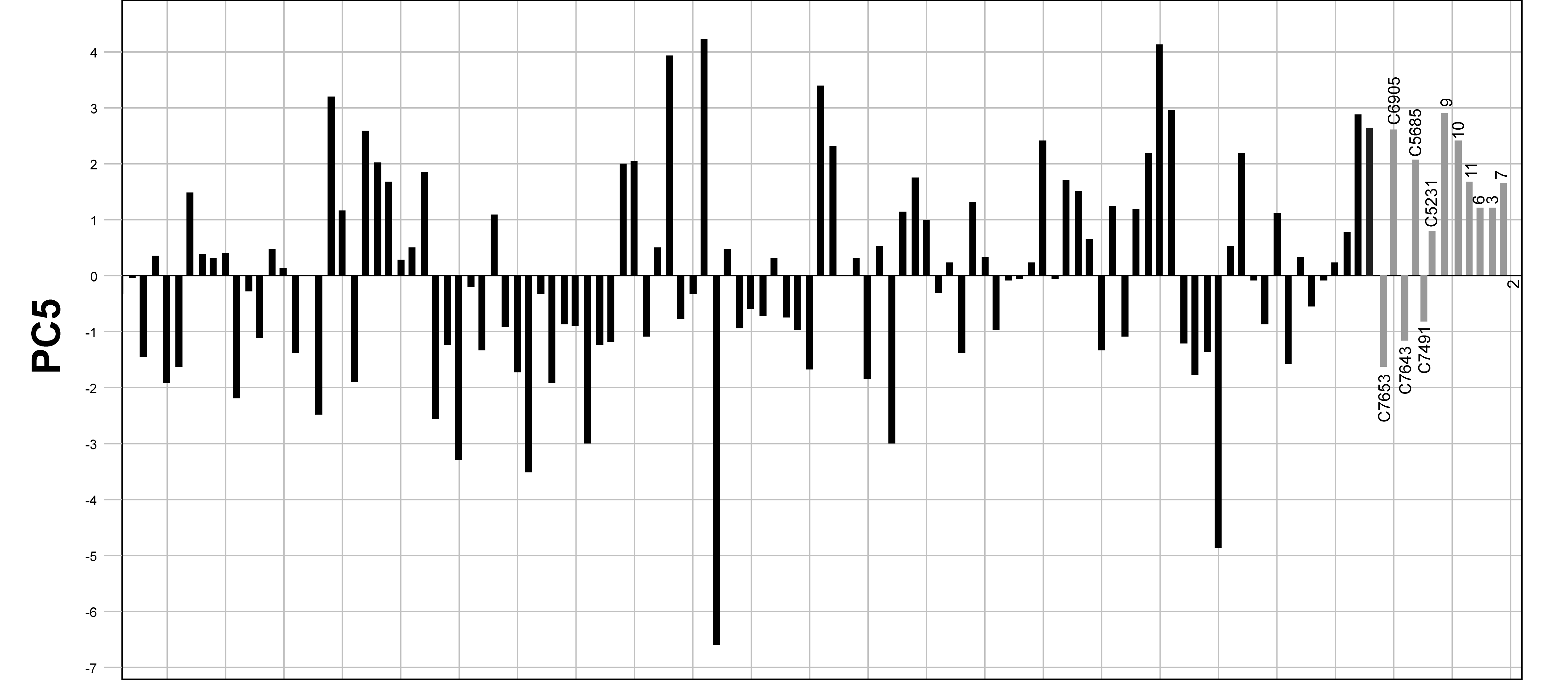


**Figure S2.** The fifth PC of the chemical space established by PCA of the physicochemical properties of the identified AChE inhibitors. The grey bars represent the hits for which crystal structures were successfully determined as well as a selection known inhibitors of AChE that were projected into the established chemical space. The compound IDs are in agreement with Figure 1 and Table 2 in the main text.


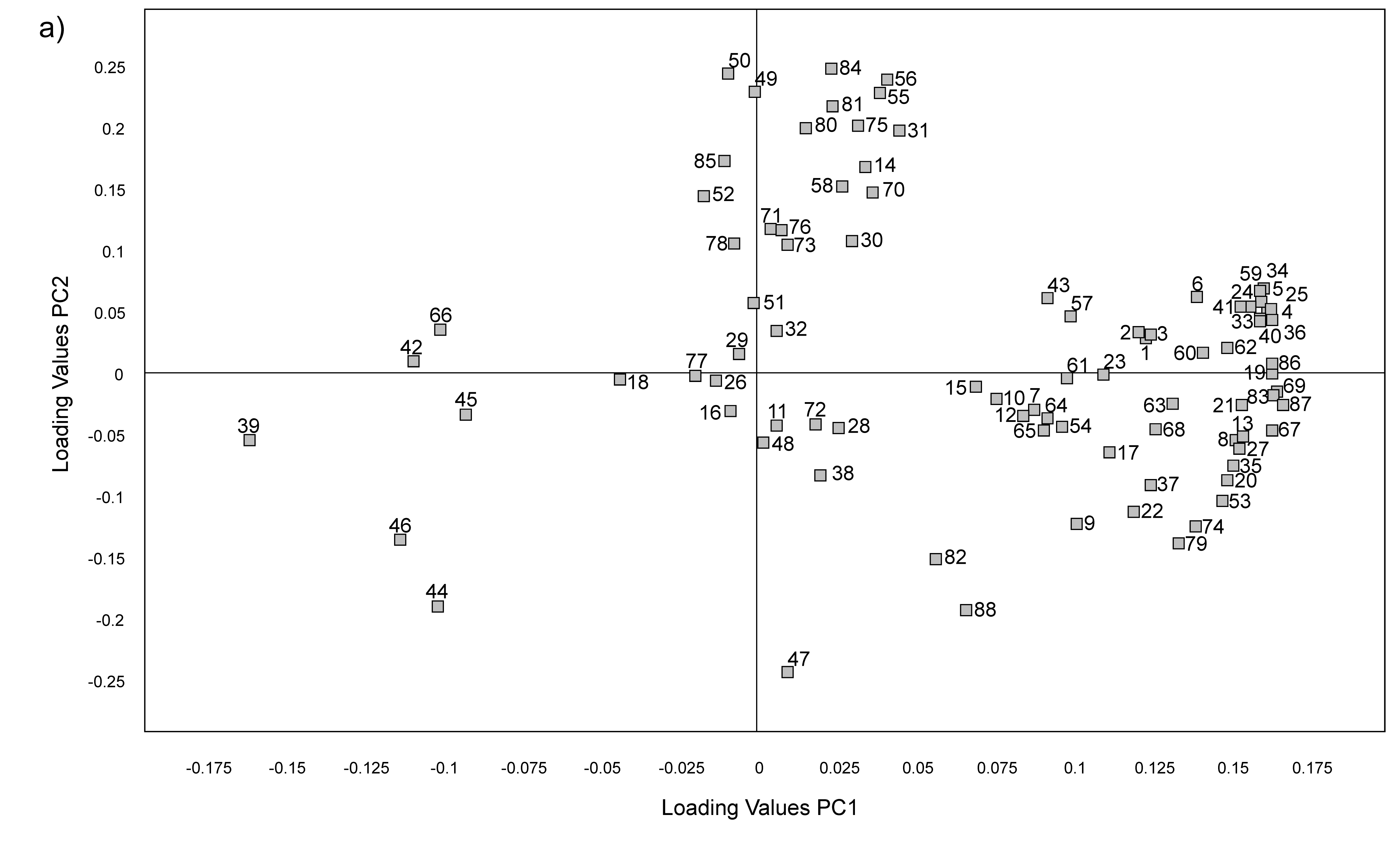

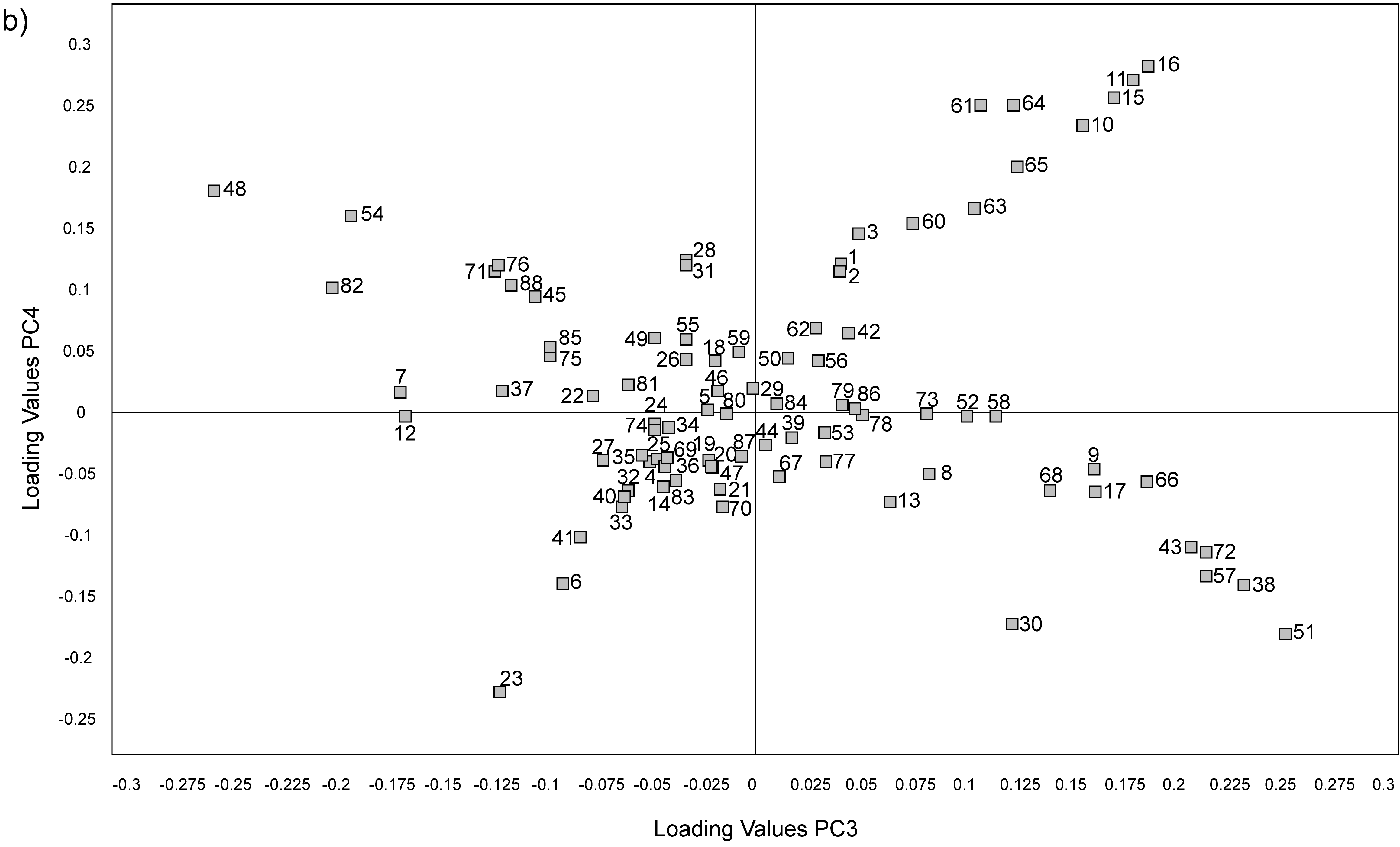

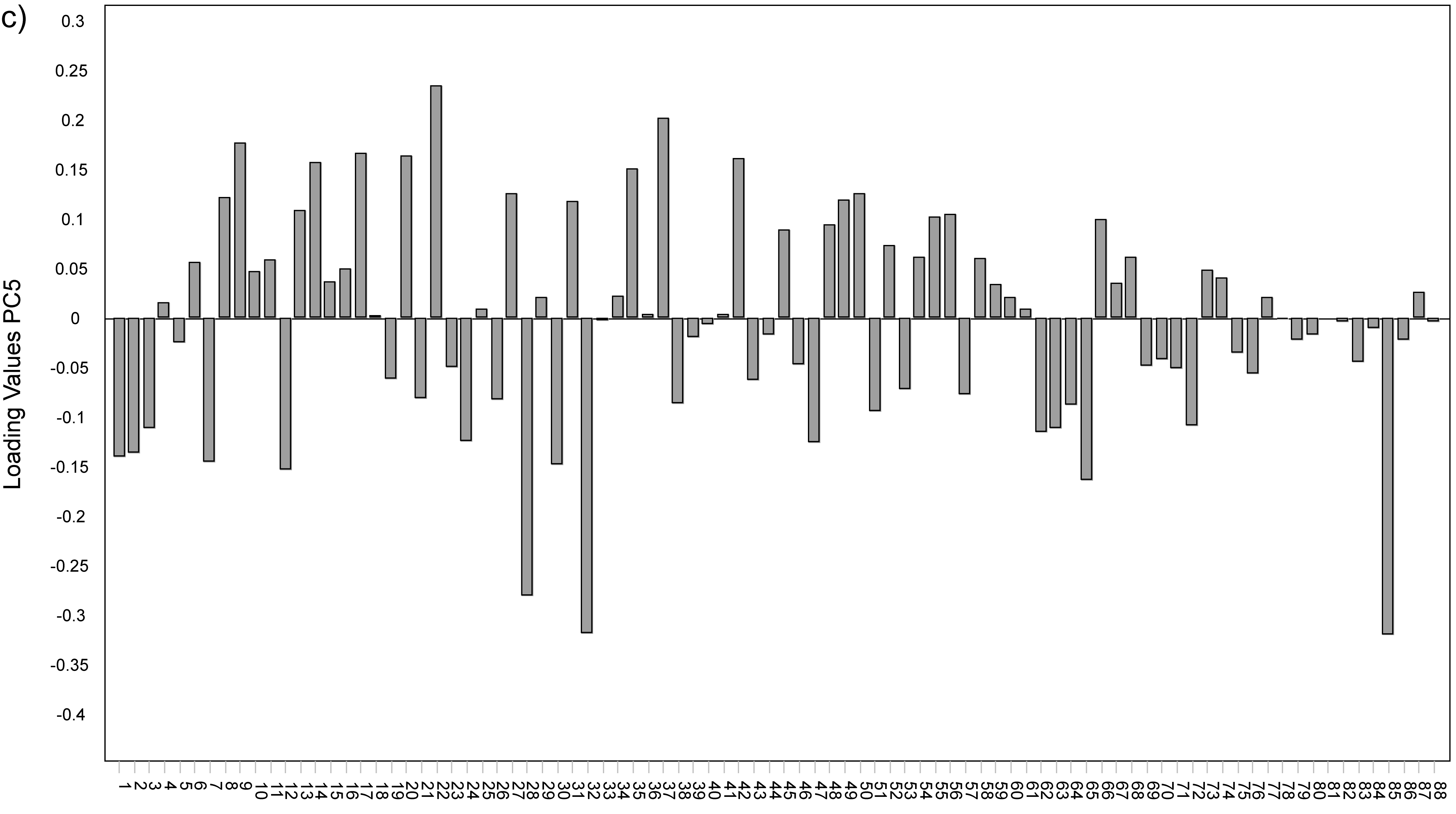


**Figure S3.** PCA loading plots of (a) PC1 versus PC2, (b) PC3 versus PC4, and (c) PC5. The molecular descriptors included in the model are indicated with the numbers assigned in Table S1.


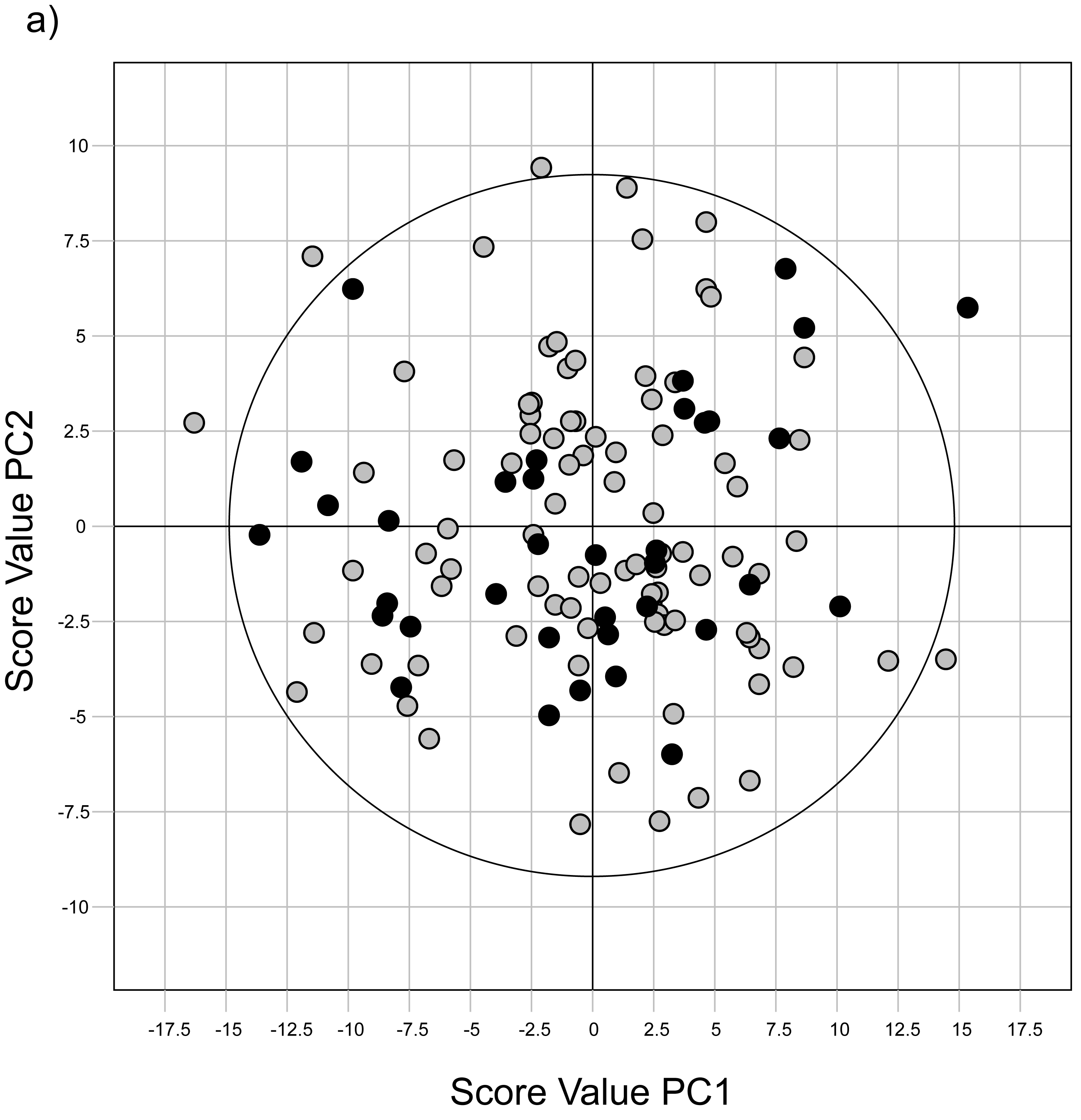


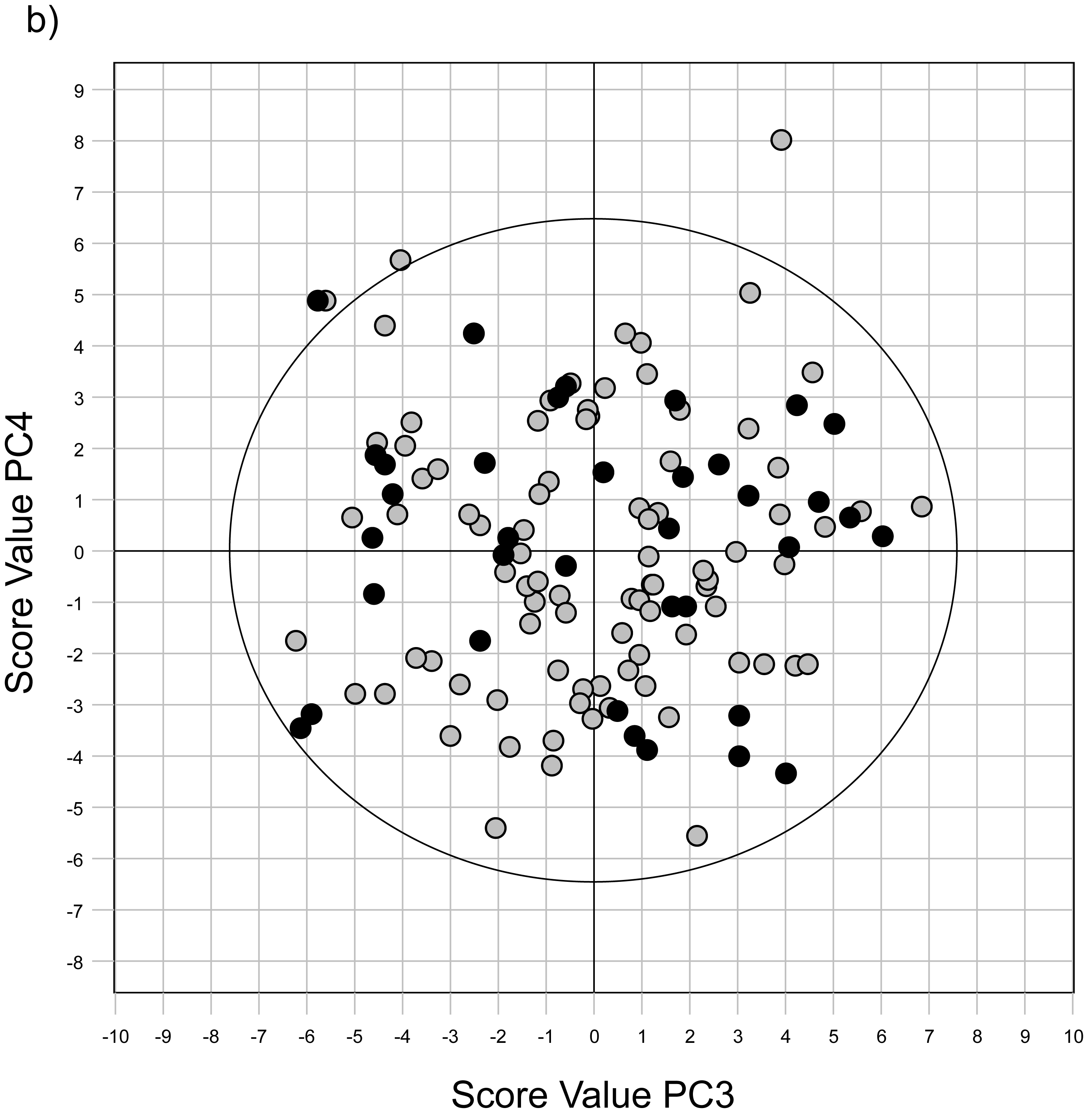
**Figure S4.** PCA score plots for (a) PC1 versus PC2, and (b) PC3 versus PC4. The hits used in crystallization trials are shown as black dots.


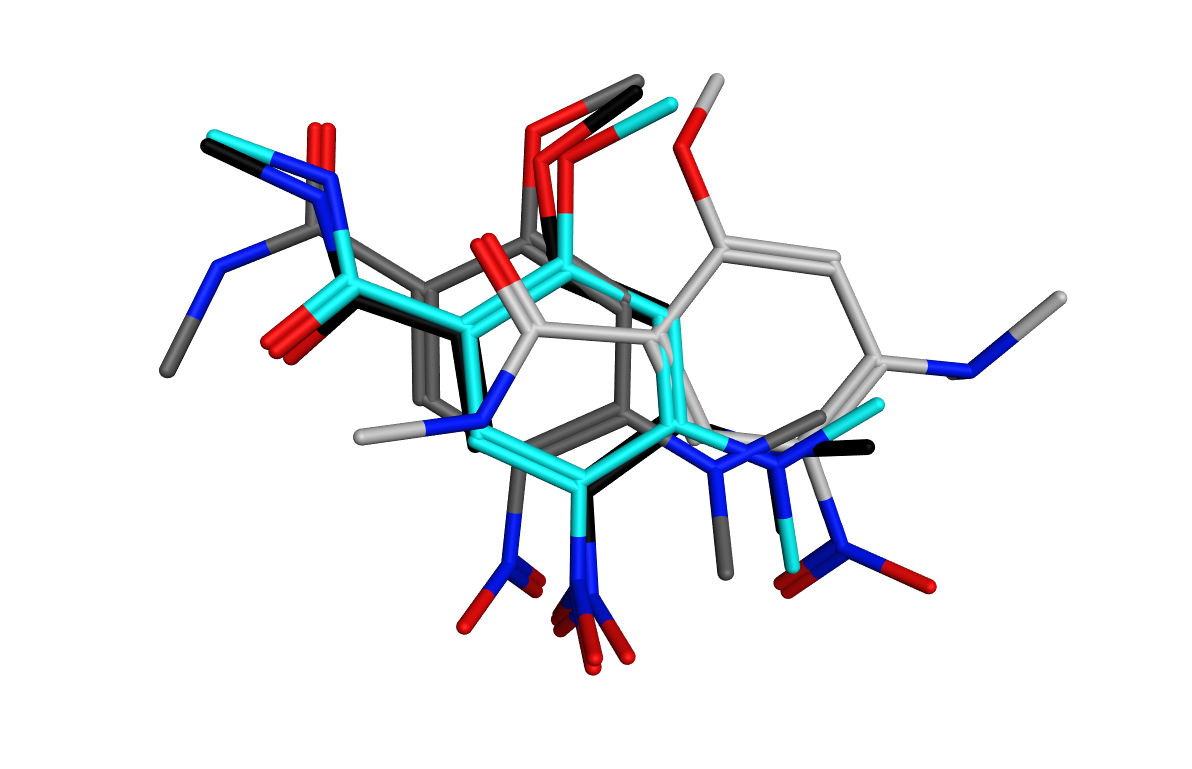


**Figure S5.** Overlay of C5685 docking poses with the X-ray crystal ligand. The poses have been truncated according to the part of the ligand modelled in the X-ray crystal structure, here shown with carbons coloured in cyan. The carbons of the poses are color-coded according to RMDS: black represents a low RMSD pose (0.48 Å), dark grey a moderate RMSD pose (1.35 Å) and light grey a high RMSD pose (2.97 Å).
